# Working from home during the COVID‐19 crisis: How self‐control strategies elucidate employees' job performance

**DOI:** 10.1111/apps.12352

**Published:** 2021-11-04

**Authors:** Eve Sarah Troll, Laura Venz, Fritzi Weitzenegger, David D. Loschelder

**Affiliations:** ^1^ Department of Psychology University of Siegen Siegen Germany; ^2^ Institute of Management and Organization Leuphana University of Lüneburg Lüneburg Germany; ^3^ Department of Psychology University of Potsdam Potsdam Germany

**Keywords:** COVID‐19, process model, self‐control strategies, telework, trait self‐control

## Abstract

Employees around the globe experience manifold challenges to maintain job performance during the so‐called work‐from‐home experiment caused by the COVID‐19 crisis. Whereas the self‐control literature suggests that higher trait self‐control should enable employees to deal with these demands more effectively, we know little about the underlying mechanisms. In a mixed‐methods approach and two waves of data collection, we examine how self‐control strategies elucidate the link between teleworking employees' trait self‐control and their job performance. Using a qualitative approach, we explored which strategies employees use to telework effectively (*N* = 266). In line with the process model of self‐control, reported strategies pertained to situation modification (i.e., altering the physical, somatic, or social conditions) and cognitive change (i.e., goal setting, planning/scheduling, and autonomous motivation). Subsequent preregistered, quantitative analyses with a diverse sample of 106 teleworkers corroborated that higher trait self‐control is related to job performance beyond situational demands and prior performance. Among all self‐control strategies, modifying somatic conditions and autonomous motivation was significantly associated with job performance and mediated the self‐control‐performance link. This research provides novel insights into the processes by which employees productively work from home and inspires a broad(er) view on the topic of self‐control at work.

## INTRODUCTION

On January 30, 2020, the World Health Organization declared the COVID‐19 outbreak a public health crisis of global concern (World Health Organization, 2020). Since then, the pandemic has confronted employees worldwide with a novel and unique situation. As countries implemented lockdown measures to contain the spread of the virus, companies around the globe sent their employees to work from home—many for the first time. Indeed, 88 per cent of employees teleworked regularly during the pandemic (Iometrics and Global Workplace Analytics, 2020), whereas only 31 per cent had regularly worked from home before. Not surprisingly, scholars have referred to this situation as the “work from home experiment” (Kramer & Kramer, 2020, p. 1).

This unprecedented shift to telework introduced manifold challenges for employees while seeking to maintain their job performance. These challenges range from creating a workplace at home to establishing new structures in working with colleagues to navigating the work–nonwork interface as many took care of their children during work hours, with schools and kindergartens closed. Such demands led to a decrease in employees' well‐being (e.g., Zacher & Rudolph, 2021). However, the pronounced demands in times of the COVID‐19 crisis, such as demands resulting from the blurriness of the work–family boundary (Shao et al., 2021), likely also pose constant challenges for employees' job performance (e.g., Gilboa et al., 2008). Corroborating this assumption, many employees reported to be less productive when working from home during the pandemic (e.g., Zacharakis & Loos, 2020). These observations place the following research questions center stage: How do employees deal with the demands that arise from teleworking? What predicts employees' ability to work from home productively and to master the novel challenges of teleworking?

The present study addresses these questions qualitatively and quantitatively. In the qualitative part of our study, we explore various self‐control strategies that employees report to use when dealing with demands related to teleworking. We build on the process model of self‐control (Duckworth et al., 2014) to organise the various strategies according to the point in time at which they are implemented—such as do employees modify their telework situation from the beginning or do they change their thoughts about the situation when in the middle of it? Building upon and extending our qualitative findings, we use a preregistered, quantitative approach to examine the role of these self‐control strategies in predicting telework outcomes. Specifically, we propose that self‐control strategies can empirically explain the link between trait self‐control (i.e., employees' ability to override impulses and to engage in potentially aversive activities; Carver, 2019) and employees' respective job performance when working from home.

The present research seeks to expand the conceptual, theoretical, and practical understanding of the experience of working from home in two crucial ways: First, prior to the pandemic, only an exclusive group of employees had the opportunity to work from home, for instance, higher income and “white‐collar” workers (Desilver, 2020). The lockdown measures, however, resulted in a much broader range of employees working from home (e.g., Pauly & Holdampf‐Wendel, 2020). Thereby this work‐from‐home experiment offers the unique opportunity to advance the understanding of which employees work more effectively from home than others (Kramer & Kramer, 2020). In this regard, we examine whether trait self‐control qualifies as a key psychological antecedent for employees' teleworking performance. Second, we examine the processes by which employees deal with the demands and challenges of teleworking and navigating the work–nonwork interface. Prior organisational research has predominantly focused on only one component of self‐control in the work domain (Lian et al., 2017): Individuals seem to enter a state of diminished self‐control resources after initial effortful acts of self‐control and, as a result, fail in self‐control subsequently—the so‐called ego depletion phenomenon (Baumeister et al., 1998; Muraven et al., 1998). For instance, employees consumed more unhealthy snacks and police employees showed less physical activity on days with more self‐regulatory demands (Sonnentag et al., 2017; Sonnentag & Jelden, 2009). In juxtaposition, other components of self‐control have received much less attention but might be equally (or even more) important (Lian et al., 2017). Examining the underlying psychological processes by which employees deal with demands of teleworking inspires a broader theoretical view on self‐control at work. Further corroborating the need for this research, plenty of anecdotal evidence has been put forward on the diverse strategies employees might use in order to work productively from home during the pandemic (e.g., Böker et al., 2020; Morgan, 2020), but so far, we lack empirical evidence on the practical use and effectiveness of such strategies.

In sum, with its unique challenges and the heterogeneous participants, the ongoing work‐from‐home experiment offers novel insights into the diverse means by which employees deal with demands related with telework during the COVID‐19 crisis and the effectiveness of applied self‐control strategies. With the present research, we seek to advance the theoretical understanding of these processes and inspire a broader perspective on self‐control at work. Thereby, we complement the previous work design perspective (Wang et al., 2021) with a focus on self‐initiated strategies that employees use to actively foster their telework performance. From a practical perspective, these insights appear more relevant than ever before: Working from home not only became unexpectedly prevalent, but it also experienced increasing appreciation by employees and employers. Many employees wish to continue teleworking after the pandemic (Stürz et al., 2020), and employers plan to foster work‐from‐home arrangements (FAZ, 2020). Illuminating the antecedents and underlying processes that help employees telework more effectively promises practical implications for the future of work.

### Trait self‐control and job performance

Whereas some employees are quite good at behaving in ways congruent with their goals, others are less successful in this endeavor. Trait self‐control^1^ describes these individual differences and can be defined as the “ability to override impulses to act, as well as the ability to make oneself initiate or persist in boring, difficult, or disliked activity” (Carver, 2019, p. 477). The benefits of higher trait self‐control are well supported: Individuals with more self‐control excel academically and professionally, are physically healthier, have better social relationships, and are less prone to unemployment (Daly et al., 2015; de Ridder et al., 2012; Duckworth & Seligman, 2005; Moffitt et al., 2011; Tangney et al., 2004). Recent research also shows that individuals higher in trait self‐control adhere more to social distancing rules during the COVID‐19 pandemic (Wolff et al., 2020). Surprisingly, little is known, however, about *how* and *why* individuals with more trait self‐control attain their (work) goals more effectively.

We build on prior self‐control theorizing to shed light on these underlying mechanisms. A variety of models and theories seek to explain the many advantageous effects of self‐control (see Inzlicht et al., 2020, for a recent review). For decades, psychological (Baumeister & Vohs, 2016) and organisational research (Lian et al., 2017) focused on ego depletion. To explain ego depletion, the prominent strength model of self‐control (Baumeister et al., 1998) suggests that exerting self‐control drains a central, nonspecific, yet limited resource. After initial effortful acts of self‐control, this resource is depleted and self‐control failure becomes more likely. This led to the (sometimes implicit) assumption that individuals *effortfully* inhibit unwanted impulses or undesired behavior when enacting self‐control (Fujita, 2011)—for instance, when suppressing the urge to take a short break from a demanding work task. The lay term “willpower” similarly implies that people need to exert deliberate effort to resist undesirable impulses to stay productive (Galla & Duckworth, 2015). Based on these notions, the central approach to explain self‐control benefits has been that individuals with high(er) trait self‐control are simply better in effortfully inhibiting impulses. However, the current discussion around the conceptualization of self‐control stresses that this effortful inhibition might be overrated (Inzlicht & Friese, 2021). Paradoxically, individuals high(er) in trait self‐control have reported *less* inhibition of impulses (Hofmann et al., 2012; Imhoff et al., 2014). It seems that by focusing only on one component of self‐control, the literature “has failed to appreciate the broader picture of what components are involved in the self‐control process” (Lian et al., 2017, p. 703).

To advance this broader picture, scholars have proposed alternative models to explain the underlying mechanisms of self‐control (e.g., Hofmann et al., 2009; Kurzban et al., 2013). We theoretically build on the process model of self‐control (Duckworth et al., 2014; Duckworth, White, et al., 2016) to illuminate how employees master the work‐from‐home experiment. Considering the entire process of enacting self‐control, the model articulates how impulses emerge (e.g., the impulse to take a break from working) and the diverse ways in which individuals can regulate these impulses. The model assumes that a multiplicity of self‐control strategies helps individuals to make themselves initiate or persist in (potentially aversive) activities. The inhibition of impulses in the heat of the moment is only the very last resort. Our research builds on this process model of self‐control to examine different mechanisms by which employees with high(er) self‐control foster desirable and prevent undesirable behaviors (i.e., impulses) to ultimately attain their work‐related goals more effectively.

### Self‐control strategies at work

**FIGURE 1:**
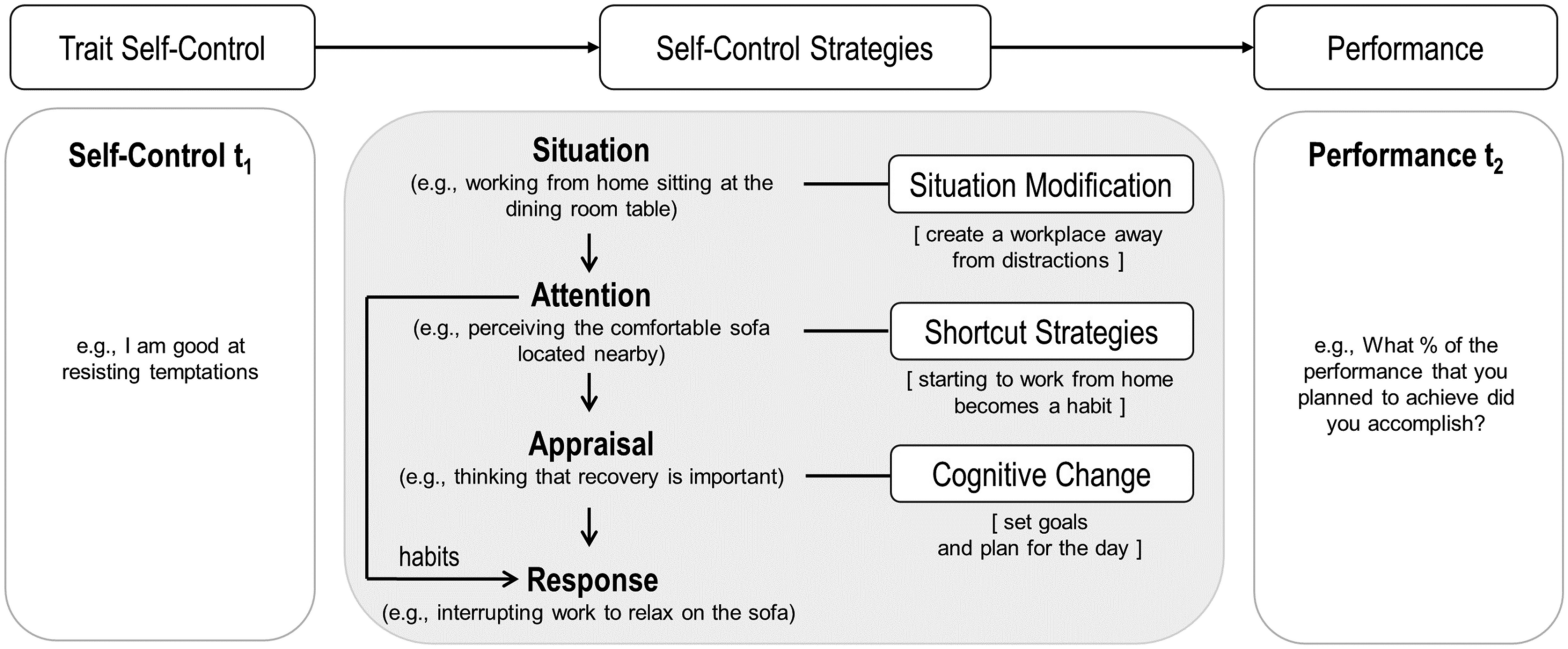
Hypothesized model. Within the process of enacting self‐control, self‐control strategies intervene and disrupt the execution of unwanted impulses (gray box; see Duckworth et al., 2014). We hypothesize that trait self‐control predicts self‐control strategy use that in turn predicts job performance

Drawing from the process model of emotion regulation (Gross, 1998), the process model of self‐control (Duckworth et al., 2014) suggests that behaviors and impulses are generated in a recursive sequence (see Figure 1): An individual encounters a particular situation, then attends to particular features of the situation, appraises the situation, and responds in a particular way. For example, an employee encounters the novel situation of working from home sitting at the dining room table, then perceives the comfortable sofa located nearby, appraises that recovery is important, and interrupts working to relax on the sofa. This process repeats itself, but now the employee is already relaxing on the sofa, which strengthens the impulse to stay there. Self‐control strategies can be organised according to the stage at which they seek to thwart a self‐control challenge (see Figure 1; Duckworth et al., 2014).

*Situation modification* strategies occur at an early stage and entail positively changing the circumstances of a current activity in ways that make it easier to initiate the activity or to persist in it (Duckworth, Gendler, & Gross, 2016). According to process model theorizing (Duckworth, Gendler, & Gross, 2016), situation modification strategies can principally be applied to physical, somatic, or social conditions of a situation. Thus far, however, empirical research has predominantly focused on the modification of the physical situation. For instance, individuals higher in trait self‐control report to minimize distractions in their environment to attain their long‐term goals (Ent et al., 2015; Hennecke et al., 2019). Corroborating the effectiveness of such physical situation modification, individuals are more likely to achieve their goals when instructed to remove temptations that might distract them from reaching their goal compared with individuals who use sole willpower or no strategy at all (Duckworth, White, et al., 2016). For the teleworker, modifying the physical conditions might mean to create a workplace as far away as possible from sofas and other temptations.

Sometimes employees face situations, however, which they cannot modify. For example, the employee forced to work from home might simply lack the space to work out of sight of their sofa (Allen et al., 2021). Here *cognitive change* strategies may help, which entail thinking about a given situation differently (Duckworth, Gendler, & Gross, 2016). These strategies seek to make long‐term choices more appealing and feasible. For instance, goals represent what people hope to accomplish. The effectiveness of goal setting (Epton et al., 2017; Locke & Latham, 2002) and planning to attain goals (Gollwitzer & Sheeran, 2006) is well‐supported empirically. Further, individuals higher in trait self‐control are more likely to make plans (Sjåstad & Baumeister, 2018). For the teleworker, cognitive change might mean to set specific goals for the day, which the employee can incrementally work towards step by step.

In addition to these intermediate stages of impulse generation, *shortcut strategies* directly link situational features to desired responses (Duckworth et al., 2019). Habits constitute such a shortcut strategy. They are executed unconsciously and thereby circumvent the appraisal stage altogether (Neal et al., 2012). Habits are developed through repeated enactment of certain activities under stable conditions. After several repetitions, behavior is initiated automatically by situational cues (see Wood, 2017; Wood & Rünger, 2016, for reviews). Research shows that individuals higher in trait self‐control rely on habits to eat more healthily, to exercise more regularly, and to perform better academically (Adriaanse et al., 2014; Galla & Duckworth, 2015; Gillebaart & Adriaanse, 2017). For the teleworker, an effective shortcut strategy might be that, after several repetitions, starting to work from home at 8:00 a.m. in the morning at the distraction‐free workplace becomes a habitual behavior.

In sum, the current discussion around the conceptualization of self‐control suggests that the effortful inhibition of (unwanted) impulses in the heat of the moment might be overrated (Inzlicht & Friese, 2021). Instead, there exists a wide range of less effortful self‐control strategies that individuals spontaneously use in their everyday life (Hennecke et al., 2019), ranging from proactively changing their situation (e.g., Ent et al., 2015) to establishing beneficial habits (e.g., Galla & Duckworth, 2015). However, so far, little is known about which self‐control strategies employees implement to pursue their work‐related tasks and how effectively they do so.

### research question and hypotheses

Previous research on self‐control at work largely focused on effortful inhibition and the ego depletion phenomenon, whereas other components of self‐control have received little attention (Lian et al., 2017). In the present research, we assume a broad(er) perspective on self‐control at work and seek to shed light on the diverse means by which employees deal with the manifold demands related to telework during the COVID‐19 crisis. We qualitatively explore the use and quantitatively examine the effectiveness of these applied self‐control strategies. In all, expanding the present literature, we examine which self‐control strategies employees use to work productively from home, how these strategies can be integrated in the process model of self‐control, and how each empirically illuminates a potential productivity advantage of employees with higher trait self‐control. First, following a qualitative approach, we examine:

Research Question 1 Which self‐control strategies do employees report to use to work productively from home?

Second, using a quantitative approach, we seek to replicate the established self‐control‐performance link in this unique telework situation by examining whether employees higher in trait self‐control are better able to work productively from home. We subsequently investigate whether (and which) self‐control strategies can explain this link. Of importance for the present research is that the benefits of trait self‐control are especially pronounced with regard to job performance (see de Ridder et al., 2012, for a meta‐analysis). For instance, individuals higher in trait self‐control are more likely to seek improvement after feedback (Ruttan & Nordgren, 2015) and are less likely to show delinquent behavior at work (Cohen et al., 2014). Employees often have degrees of freedom in choosing whether, how, and when to perform their tasks (Johnson et al., 2018). Thereby, they shoulder the responsibility to make themselves initiate or persist in their work duties. Self‐control is center stage as employees control impulses, resist distractions, ignore temptations, and overcome inner resistances to achieve work‐related goals (Schmidt & Diestel, 2015). In other words, maintaining job performance requires to deal with numerous self‐control demands. The sudden move to work from home as part of the of COVID‐19 lockdown measures likely led employees to experience even more pronounced decision latitude (Gajendran & Harrison, 2007) and, thus, likely introduced additional temptations and undesired impulses. As a result, many face not only the usual demands that work places on self‐control (Schmidt & Diestel, 2015) but also additional demands, distractions, and obligations in the work–nonwork interface (e.g., Shao et al., 2021). Trait self‐control should enable employees to overcome these manifold challenges (e.g., creating a workplace and childcare) more effectively in favor of their job‐related goals (Lian et al., 2017).

Hypothesis 1 Trait self‐control positively relates to teleworkers' performance over and above situational demands (e.g., separate workroom and childcare).

In addition, we expect that employees attain their goals via a multiplicity of self‐control strategies. Plausibly, individuals higher in trait self‐control are inclined to use such self‐control strategies to resist temptations and impulses in favor of their goals. Indeed, individuals higher in trait self‐control more likely use situation modification (e.g., Ent et al., 2015), cognitive change (e.g., Sjåstad & Baumeister, 2018), and shortcut strategies (e.g., Galla & Duckworth, 2015). In turn, situation modification (e.g., Duckworth, White, et al., 2016), cognitive change (e.g., Gollwitzer & Sheeran, 2006), and shortcut strategies (e.g., Galla & Duckworth, 2015) can help individuals attain their goals more effectively. Thus, we propose that trait self‐control is associated with teleworkers' use of self‐control strategies and that the strategies positively relate to their job performance.

Hypothesis 2 Trait self‐control is positively associated with teleworkers' use of self‐control strategies of (a) situation modification, (b) cognitive change, and (c) shortcut strategies.

Hypothesis 3 Self‐control strategies of (a) situation modification, (b) cognitive change, and (c) shortcut strategies positively predict teleworkers' job performance.

In sum, we predict that the link between trait self‐control and teleworkers' job performance is mediated via self‐control strategies (see Duckworth et al., 2014; Gross, 1998, for a similar reasoning). Self‐control strategies lend themselves as a plausible mechanism by which unobservable trait self‐control manifests as observable behavior.

Hypothesis 4 The positive association between trait self‐control and teleworkers' job performance is mediated through the use of self‐control strategies of (a) situation modification, (b) cognitive change, and (c) shortcut strategies.

## METHODS

This preregistered study included two waves of data collection, one at the beginning of April 2020 (t_1_) and the second at the end of April 2020 (t_2_). At t_1_, our German sample of employees had just entered lockdown. At t_2_, they had spent about a month in lockdown working from home. The preregistration (https://osf.io/yw8ma), data, and an analysis script are publicly available on the Open Science Framework (https://osf.io/9egkb/). We recruited German‐speaking employees via social networks and email contacts from regional companies that we have collaborated with in the past. We deliberately recruited a broad sample of experienced employees from diverse sectors and professions (e.g., consultants, administrative staff, therapists, academics, engineers, and social workers). Those who indicated that they were currently not working from home were thanked for their interest and forwarded to the last page of the online questionnaire. Participants first provided open text answers describing which strategies they use to work productively from home and then completed the quantitative survey part. At t_1_, participants also provided demographic information and their email address to contact them for the t_2_ questionnaire. Participants at t_1_ were 266 employees who currently worked from home (72.18% female, 27.44% male, 0.38% diverse; *M*_age_ = 37.00 years, *SD* = 11.91). The qualitative part of our study is based on this sample.

After 3 weeks, we contacted all participants who provided their email address at t_1_ (*N* = 181) and asked them to participate in the second questionnaire. A reminder was sent 3 days later. In total, 128 participants completed both questionnaires. To examine selective dropout, we contrasted trait self‐control between employees who participated only at t_1_ (*M* = 4.60, *SD* = 0.92) with those who participated at t_1_ and t_2_ (*M* = 4.73, *SD* = 0.91). We found no significant difference, *t*(252) = −1.13, *p* = .258, suggesting no selective dropout. Following our preregistered exclusion criteria, we excluded nine participants who indicated to not work from home at t_2_, three participants who did not pass the attention check (“Please select the answer option on the far left”; Oppenheimer et al., 2009), and ten participants whose scores exceeded > 2.5 *SD*s from the overall mean. This resulted in a final sample of 106 participants that we used for the quantitative part of our study.^2^ The sample consisted of employees (83.96%), working students (11.32%), and self‐employed individuals (4.72%). Monthly net incomes ranged from < €1000 (11.32%), < €2000 (33.02%), < €3000 (32.08%), and < €4000 (11.32%) to > €4000 (10.38%). Mean (contract) working hours were 31.81 h (*SD* = 8.92).

## QUALITATIVE PART: EXPLORATORY ANALYSIS OF SELF‐CONTROL STRATEGIES

We followed a qualitative approach to explore which strategies employees report to use to productively telework during the COVID‐19 crisis. Participants answered the question “What do you do to work productively from home?” in an open response format. Participants reported between 0 and 7 different strategies (total of 480 valid responses). In line with prior research (Hennecke et al., 2019), we used a multiple‐step procedure to derive categories of self‐control strategies from these responses. First, one member of our research team identified categories based on a sample of 100 randomly selected responses. Subsequently, a research assistant used these categories to code the same 100 responses to estimate the categories' reliability. We then coded all 480 responses to determine the prevalence of each strategy.

### Results of the qualitative part

The random sample of 100 responses produced six categories of self‐control strategies. We calculated interrater reliability using *kappa* (κ) for these categorical data (Cohen, 1968). *κ* = .82 suggested high reliability according to common standards (Cicchetti, 1994). Table 1 lists all categories, detailed descriptions, verbatim examples, and prevalence. The identified categories reflected the theoretical assumptions of the process model of self‐control (Duckworth et al., 2014). Specifically, three categories emerged that referred to altering employees' *physical environment* (e.g., “minimize distractions”), *somatic conditions* (e.g., “dressing up as for the office”), and *social conditions* (e.g., “contact and agreements with colleagues”). Each of these strategies changes the situational circumstances in ways that make it easier for individuals to initiate an activity (e.g., start working on a task) or to persist in it, while leaving the activity itself unchanged (Hennecke et al., 2019). Thus, with these different substrategies, teleworkers foster different kinds of resources (i.e., physical, somatic, or social), which might improve job performance (Bakker & Demerouti, 2017). *Goal setting* (e.g., “set achievable goals”), *planning/scheduling* (e.g., “determine times for leisure”), and *autonomous motivation* (e.g., “make me aware of the importance of the activity”) can be subsumed as strategies of cognitive change. Each of these strategies seeks to change one's cognitive thoughts about a given situation in ways that make an activity appear more appealing and feasible (Hennecke et al., 2019).

**TABLE 1:** Self‐control strategies, descriptions, verbatim examples, and prevalences from the qualitative analysis

Strategy	Description	Verbatim examples	Prevalence
Situation modification			54.21%
a. Physical condition	Build an advantageous environment (e.g., by providing necessary materials or by reducing distractions)	“Minimize distractions.” “Put on my headphones.” “Bring materials from my company.”	29.59%
b. Somatic condition	Modify one's somatic environment advantageously (e.g., by taking substances or by changing one's physical state)	“Drink coffee.” “Let fresh air in.” “Dressing up as for the office.”	15.55%
c. Social condition	Use the support of and the commitment to others (e.g., by meeting regularly with colleagues)	“Tell others in my household that I'm working.” “Contact and agreements with colleagues.”	9.07%
Cognitive change			45.79%
d. Goal setting	Set and commit to (sub)goals (e.g., by defining specific goals and corresponding deadlines)	“Set achievable goals.” “To‐do lists to check off.”	15.12%
e. Planning/scheduling	Make or commit to a schedule or routine (e.g., by sticking to a daily structure including breaks)	“Maintain structures from the office at home.” “Determine times for leisure.”	29.37%
f. Autonomous motivation	Motivate oneself to initiate or persist in an activity (e.g., by reminding oneself of the relevance of the activity)	“Make me aware of the importance of the activity.”	1.30%

*Note*: Self‐control strategies were derived from open answers of the total sample of *N* = 266 with a sum of 480 responses. A total of 17 responses could not be clearly assigned to one of those categories (see the supplemental online material on https://osf.io/9egkb/).

Of the six mentioned categories, altering the physical environment (29.59%) and planning/scheduling (29.37%) were named most frequently, followed by altering one's somatic condition (15.55%), goal setting (15.12%), and altering social conditions (9.07%). Only a small share of responses (1.30%) referred to fostering autonomous motivation in employees' daily work. A total of 17 responses could not be clearly assigned to one of the six self‐control strategy categories (see the supplemental online material on https://osf.io/9egkb/): Some answers did not clearly refer to behaviors to *foster* productive telework (e.g., “playing Xbox” or “distraction through WhatsApp and social media”), and other responses could not be clearly assigned to one of the more prevalent categories (e.g., “discipline”).

### Interim discussion

The qualitative analysis aimed at identifying self‐control strategies that employees report to use to work from home productively. Although these results likely do not reflect an exhaustive list of self‐control strategies, they cover the strategies that employees explicitly considered relevant and helpful. Employees' responses corroborated the theoretical assumptions of the process model of self‐control (Duckworth et al., 2014): three forms of situation modification (Duckworth, Gendler, & Gross, 2016) emerged—employees reported to alter the physical (e.g., minimize distraction), somatic (e.g., sleep, fresh air, and coffee), and social conditions (e.g., stay in touch with colleagues) of their work situation. In line with the focus of previous research (e.g., Ent et al., 2015), employees most often reported to change their physical circumstances; however, considerable shares also referred to changing somatic and social conditions of the current working situation. These qualitative findings indicate that each of these aspects of situation modification may be important—not physical situation modification alone. With regard to cognitive change strategies (Duckworth et al., 2014), we identified goal setting, planning/scheduling, and fostering autonomous motivation as conducive strategies with the latter being mentioned rarely, however. Goal setting and planning/scheduling have been previously identified as self‐control strategies in the literature (Hennecke et al., 2019). Although previous research points to the potentially crucial role of autonomous motivation in goal pursuit (Werner & Milyavskaya, 2019), the strategy to actively foster one's autonomous motivation (e.g., “make me aware of the importance of the activity”) is reported only by a small share of employees. None of the responses referred to habit automaticity as a form of shortcut strategy, which may be explained by the fact that these behaviors are executed rather unconsciously (Neal et al., 2012). In sum, our qualitative analysis shows that employees use a wide range of self‐control strategies while working from home. However, this does not necessarily mean that each strategy helps employees to pursue their work‐related tasks more effectively. The following quantitative part thus examines the effectiveness of the established self‐control strategies.

## QUANTITATIVE PART: SELF‐CONTROL STRATEGIES AS MULTIPLE MEDIATORS

The quantitative analyses investigate the association of teleworking employees' trait self‐control with (a) their use of different self‐control strategies and (b) the strategies' relative effectiveness in bolstering employees' job performance while working from home.

### Measures

We assessed employees' trait self‐control at t_1_ and the degree to which they engaged in different self‐control strategies at t_2_. Items were accompanied by 7‐point scales ranging from 1 = *strongly disagree* to 7 = *strongly agree*. We assessed job performance in both questionnaires. Table 2 shows descriptive statistics and bivariate correlations of all measures. As indicators of reliability, we report Spearman–Brown for two‐item scales (Eisinga et al., 2013) and Cronbach's α and Coefficient *H* (Hancock & Mueller, 2001) for scales with more than two items. Cronbach's α assumes that each item contributes equally to the total scale score, whereas Coefficient *H* measures maximal reliability for an optimally weighted scale, for which each item contributes different amounts of information (McNeish, 2018).

**TABLE 2:** Descriptive statistics and bivariate correlations of model variables

	*M*	*SD*	1	2	3	4	5	6	7	8	9	10	11	12	13	14
1. Trait self‐control	4.61	0.87														
Situation modification																
2. Physical conditions	5.00	1.61	.26*													
3. Somatic conditions	5.76	1.05	.32**	.41**												
4. Social conditions	5.23	1.37	.17	.22*	.29*											
Cognitive change																
5. Goal setting	4.97	1.12	.25*	.31*	.25*	.56**										
6. Planning/scheduling	4.94	1.56	.43**	.68**	.38**	.20*	.44**									
7. Autonomous motivation	5.47	1.03	.26*	.42**	.38**	.26*	.29*	.42**								
Shortcut strategy																
8. Habit	4.67	1.49	.34**	.30*	.24*	.09	.22*	.40**	.37**							
Situational demands																
9. Size of household^a^	3.08	1.34	.01	.06	.07	.03	.01	.06	.07	.08						
10. Children in household^b^	0.75	1.18	.08	−.29*	.11	.01	−.03	−.11	.04	−.11	.53**					
11. Childcare provided^c^	3.78	1.94	−.01	.26*	−.07	−.06	.04	.14	−.08	.03	−.41**	−.81**				
12. Separate working room^d^	0.50	0.50	.14	.40**	.12	.11	.17	.30*	.14	.20*	.31*	.06	−.01			
13. Working hours recorded^d^	0.36	0.48	.04	.12	.04	−.01	−.06	.12	.09	.16	.00	.04	−.07	−.08		
Performance																
14. Performance (t_1_)^e^	86.10	12.09	.37**	.41**	.20*	.21*	.22*	.40**	.37**	.29*	.11	−.01	.03	.15	.30*	
15. Performance (t_2_)^e^	85.25	14.30	.32**	.44**	.48**	.27*	.29*	.42**	.49**	.34**	.08	.00	−.05	.19	.20*	.46**

*Note*: *N* = 106. t_1_ = first measurement point. t_2_ = second measurement point.

^a^ 1 = less than 50 m^2^; 2 = less than 80 m^2^; 3 = less than 100 m^2^; 4 = less than 150 m^2^; 5 = less than 200 m^2^; 6 = more than 200 m^2^.

^b^ 0 = there are no children in my household; 1 = one child; 2 = two children; 3 = three children; 4 = more than three children.

^c^ 0 =  never; 1 = rarely; 2 =  now and then; 3 =  sometimes; 4 = always; 5 =  there are no children in my household.

^d^ 0 = no; 1 = yes.

^e^ 0 to 100 per cent.

* *p* < .05.

** *p* < .001.

#### Trait self‐control

Participants completed the German trait self‐control scale (13 items; Bertrams & Dickhäuser, 2009; Tangney et al., 2004). Sample items are “I am good at resisting temptation” and “People would say that I have iron self‐discipline” ( α = .83; *H* = .88).

#### Self‐control strategies

According to process model theorizing (Duckworth, Gendler, & Gross, 2016), situation modification strategies can be applied to physical, somatic, or social circumstances of a situation. In order to capture this range of possible situation modification strategies, we developed a measure that captures each of these aspects with one item each. The item for *physical conditions* reads “I create a workplace where I can work without interruptions and distractions”, the item for *somatic conditions* is “I put myself in a condition that allows me to work productively (e.g., putting on fresh clothes, sleeping sufficiently)”, and the item for *social conditions* reads “I have regular contact with friends and colleagues who motivate me to work productively”. Initially, we had planned to combine these three items into one overall situation modification strategy scale. However, the qualitative part of the study established that physical, somatic, and social modification strategies each make up for a large share of individuals' open responses on how to effectively work from home. Thus, we decided to account for the process model theorizing as well as participants' qualitative responses by examining the three aspects as separate strategies in the quantitative analyses.

As strategies of cognitive change, we considered goal setting, planning/scheduling, and autonomous motivation. We assessed *goal setting* with three items (“I set myself clearly defined goals for my daily work”, “I set myself specific tasks as a goal, which I then work through”, and “I set my work goals together with colleagues or friends”; *α* = .67; *H* = .82^3^) to capture different aspects of goal setting, which have been shown to be particularly effective in previous research (Epton et al., 2017; Locke & Latham, 2002). Two items assessed *planning/scheduling* (Hennecke et al., 2019) with regard to one's workday (“My working day at home is clearly structured” and “I have clear routines in my daily work”; Spearman–Brown = .89). We adapted two items used in previous research (Converse et al., 2019) to assess individuals' *autonomous motivation* while working from home (“I enjoy working from home” and “I am working from home on tasks that I consider important”; Spearman–Brown = .54).

As a shortcut strategy, we assessed *habits* using the four‐item automaticity subscale of the Self‐Report Habit Index (e.g., “I automatically put myself to work at home.”) adapted from Gardner et al. (2012; *α* = .85; *H* = .89).

#### Job performance

To assess job performance, at both t_1_ and t_2_, we asked participants to think of their previous work week. Employees rated the following three items on a slider from 0 to 100: “What % of the performance that …”: “you typically achieve at your normal workplace did you accomplish at home?” / “you could have achieved while working from home did you accomplish?” / “you planned to achieve while working from home did you accomplish?”. We let participants rate their performance in relation to these subjective standards (e.g., typical performance) in order to account for their specific working situation. Scores were averaged into a single scale to capture self‐reported job performance ( α_t1_ = .82; *H*_t1_ = .86; α_t2_ = .87; *H*_t2_ = .88).

#### Situational demands

We also assessed several situational demands relevant to working from home during the COVID‐19 pandemic—the size of one's household (in m^2^), the number of children in employees' households (counts), the degree to which childcare is provided (from 0 = *never* to 5 = *there are no children in my household*), whether participants have a separate working room (*yes* or *no*), and whether working hours are recorded (*yes* or *no*).^4^

#### Social desirability

To examine whether social desirability biases employees' responses, we assessed four items from the German Marlowe–Crowne Social Desirability Scale (Stöber, 1999): “I always openly admit my own mistakes and calmly endure any negative consequences” / “Sometimes I help only because I expect something in return” / “I have not returned borrowed items before” / “In a dispute, I always remain factual and objective”. The second and third items were recoded, and then all scores were averaged into a single scale (*α* = .44; *H* = .61). Corroborating previous research (Tangney et al., 2004), trait self‐control was positively associated with social desirability (*r* = .41, *p* < .001). Controlling for social desirability did not markedly alter the reported results; thus, we report all further analysis without social desirability as control variable.

#### Validation study

To validate our self‐developed items, we ran another study (*N* = 174) in which we asked participants from Academic Prolific (Palan & Schitter, 2018) retrospectively for their experience with working from home at the beginning of the pandemic. Based on our rich qualitative data, we created additional items to cover each self‐control strategy more broadly. To measure somatic conditions, for instance, we added three more fine‐grained items: “I made sure that I was physically fit and sufficiently rested to start the workday” / “I supplied my body with sufficient fluids and fresh air” / “I prepared myself physically for a productive workday (e.g., appropriate clothing)”. We conducted exploratory factor analysis using maximum likelihood estimation to examine the structure of each self‐developed scale and the role of the original items used in our main study in forming these multi‐item measures. Scree plots indicated a one‐factor solution for each set of items (1.32 < eigenvalues < 2.94) with uniform factor loadings (.37 < *λ* < .99). Importantly, the single items used in the main study showed consistently high factor loadings with the latent constructs (.74 < *λ* < .85) and high correlations with the added items (.50 < *r* < .80). These findings suggest that the single‐item measures used in our main study capture the respective self‐control strategy sufficiently and adequately. In addition, our self‐developed performance scale showed a high correlation (*r* = .80, *p* < .001) with adapted items from an established performance scale (e.g., “I fulfilled the responsibilities of my job”; Williams & Anderson, 1991), also corroborating the measure's validity. Please refer to the supplemental online material on https://osf.io/9egkb/ for more detailed methods and results on the validation study.

#### Confirmatory factor analysis

To examine the distinctiveness of our measures, we ran a confirmatory factor analysis using maximum likelihood estimation. A six‐factor model assuming distinct factors for trait self‐control, the multi‐item self‐control strategies, and performance ( $\chi^{2}$[309] = 438.30, *p* < .001; CFI = .876; SRMR = .076; RMSEA = .063; AIC = 11,202.78) fits the data better than alternative models, for instance, assuming one factor for all mediator items ( $\chi^{2}$[321] = 645.89, *p* < .001; CFI = .689; SRMR = .092; RMSEA = .098; AIC = 11,386.38) or one factor over all items ( $\chi^{2}$[324] = 840.23, *p* < .001; CFI = .506; SRMR = .113; RMSEA = .123; AIC = 11,574.72). Based on common conventions (Hu & Bentler, 1999), the indices of the six‐factor model indicate an “acceptable” model fit. This model is only of limited informative value, however, as it does not include measures on the three aspects of situation modification. Thus, we ran the same analysis for our validation study with multi‐item measures on each self‐control strategy. Based on common conventions (Hu & Bentler, 1999), the model indices of the resulting nine‐factor model ( $\chi^{2}$[783] = 1190.12, *p* < .001; CFI = .901; SRMR = .068; RMSEA = .055; AIC = 26,958.88) indicate a “good” model fit.

### Results of the quantitative part

We used the software R for all analyses (R Core Team, 2020). Our analysis script is available via the accompanying project on the Open Science Framework (https://osf.io/9egkb/). To test Hypothesis 1, which stated a positive relationship between trait self‐control and job performance, we conducted a multiple regression analysis investigating whether trait self‐control at t_1_ predicts performance at t_2_ beyond situational demands. As shown in Table 3, Model I, trait self‐control predicted teleworkers' job performance (*b* = 5.08, *p* = .001). The multiple regression explained 18.4 per cent of variance in performance, a small‐to‐moderate effect (Ferguson, 2009). This association remained marginally significant (*b* = 3.07, *p* = .051) when controlling for situational demands and t_1_ performance (see Table 3, Model II). Thus, Hypothesis 1 was supported.

**TABLE 3:** Multiple regression of trait self‐control (t_1_) and situational demands on performance (t_2_)

	Model I				Model II			
	*b*	95% CI	*t*	*p*	*b*	95% CI	*t*	*p*
Trait self‐control	5.08	[2.04, 8.12]	3.32	.001	3.07	[−0.02, 6.17]	1.97	.051
Size of household^a^	0.87	[−1.54, 3.29]	0.72	.474	0.34	[−1.97, 2.65]	0.29	.770
Children in household^b^	−2.91	[−6.97, 1.15]	−1.42	.158	−2.41	[−6.27, 1.45]	−1.24	.218
Childcare provided^c^	−1.41	[−3.71, 0.88]	−1.22	.225	−1.45	[−3.63, 0.72]	−1.33	.188
Separate working room^d^	4.17	[−1.37, 9.70]	1.49	.139	3.27	[−2.00, 8.55]	1.23	.221
Working hours recorded^d^	5.96	[0.58, 11.35]	2.20	.030	2.86	[−2.54, 8.26]	1.05	.300
Baseline performance (t_1_)	—	—	—	—	0.41	[0.18, 0.64]	3.50	<.001
*R* ^2^	.18				.27			

*Note*: *N* = 106.

Abbreviations: *b*, unstandardised regression coefficient; CI, confidence interval.

^a^ 1 = less than 50 m^2^; 2 = less than 80 m^2^; 3 = less than 100 m^2^; 4 = less than 150 m^2^; 5 = less than 200 m^2^; 6 = more than 200 m^2^.

^b^ 0 = there are no children in my household; 1 = one child; 2 = two children; 3 = three children; 4 = more than three children.

^c^ 0 =  never; 1 = rarely; 2 =  now and then; 3 =  sometimes; 4 =  always; 5 =  there are no children in my hoursehold.

^d^ 0 = no; 1 = yes.

To test Hypothesis 2, which stated positive relationships between trait self‐control and self‐control strategies, we examined a path model using the *lavaan* package in R (Rosseel, 2012), in which t_1_ trait self‐control predicts the seven different self‐control strategies at t_2_. To estimate practical significance of each association, we report *R* ^2^ for each endogenous variable. For instance, *R* ^2^ = .10 indicates that trait self‐control explained 10 per cent of variance in the respective variable. As Table 4 shows, trait self‐control positively predicted situation modification regarding physical (*β* =  0.26, *R* ^2^ = .07, *p* = .006) and somatic (*β* = 0.32, *R* ^2^ = .10, *p* = .001) conditions, and cognitive change, that is, goal setting (*β* =  0.25, *R* ^2^ = .06, *p* = .008), planning/scheduling (*β* =  0.43, *R* ^2^ = .18, *p* < .001), and autonomous motivation (*β* =  0.26, *R* ^2^ = .07, *p* = .005). Trait self‐control also positively predicted shortcut strategies, that is, habits (*β* =  0.34, *R* ^2^ = .12, *p* < .001). Trait self‐control did not significantly predict situation modification of social conditions (*β* =  0.17, *R* ^2^ = .03, *p* = .071). In sum, except for altering social conditions, the results support Hypothesis 2.^5^

**TABLE 4:** Path analysis from trait self‐control (t_1_) on self‐control strategies (t_2_)

	*β*_tsc_	95% CI	*R* ^2^	*p*
Situation modification				
Physical conditions	0.26	[0.07, 0.44]	.07	.006
Somatic conditions	0.32	[0.14, 0.50]	.10	.001
Social conditions	0.17	[−0.01, 0.36]	.03	.071
Cognitive change				
Goal setting	0.25	[0.06, 0.43]	.06	.008
Planning/scheduling	0.43	[0.25, 0.60]	.18	<.001
Autonomous motivation	0.26	[0.08, 0.45]	.07	.005
Shortcut strategies				
Habits	0.34	[0.16, 0.52]	.12	<.001

*Note: N* = 106.

Abbreviations: CI, confidence interval; *β*_tsc_, standardised regression coefficient from trait self‐control on the respective self‐control strategy.

To test Hypothesis 3, assuming a positive relationship between self‐control strategies and job performance, we ran a multiple regression analysis predicting t_2_ performance while controlling for t_1_ performance (*β* =  0.26, *p* = .004). Results are shown in Table 5. Of the seven self‐control strategies, only situation modification of somatic conditions (*β* =  0.28, *p* = .002) and cognitive change of autonomous motivation (*β* =  0.19, *p* = .042) showed significant, positive associations with performance. All other strategies were not significantly related to performance (.322 < *p* < .798). The multiple regression explained 44.05 per cent of variance in job performance, a moderate‐to‐strong effect (Ferguson, 2009). Hypothesis 3 was partially supported.

**TABLE 5:** Multiple regression of self‐control strategies (t_2_) predicting performance (t_2_)

	*β*	95% CI	*t*	*p*
Baseline performance (t_1_)	0.26	[0.08, 0.43]	2.92	.004
Situation modification				
Physical conditions	0.07	[−0.15, 0.29]	0.65	.518
Somatic conditions	0.28	[0.11, 0.46]	3.20	.002
Social conditions	0.03	[−0.15, 0.22]	0.37	.715
Cognitive change				
Goal setting	0.03	[−0.17, 0.23]	0.33	.744
Planning/scheduling	0.03	[−0.20, 0.26]	0.26	.798
Autonomous motivation	0.19	[0.01, 0.37]	2.06	.042
Shortcut strategies				
Habits	0.09	[−0.09, 0.26]	1.00	.322
*R* ^2^	.44			

*Note*: *N* = 106.

Abbreviations: CI, confidence interval; *β*, standardised regression coefficient.

**FIGURE 2:**
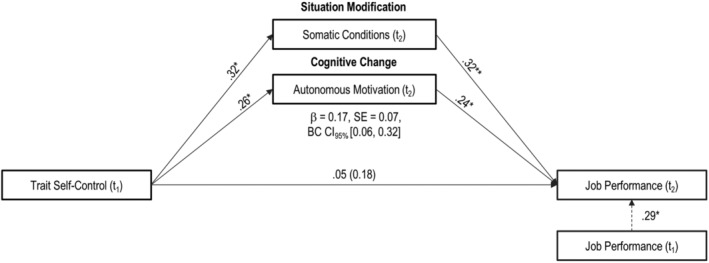
Mediation model. The multiple mediation model shows that the effect of trait self‐control at t_1_ on job performance at t_2_ is mediated by situation modification of somatic conditions and cognitive change regarding autonomous motivation. Path coefficients are standardised for both the a*‐* and b*‐*paths. Solid lines represent hypothesized associations. Dashed lines represent associations we additionally controlled for. *p < .05. **p < .001

To test Hypothesis 4, stating that self‐control strategies would mediate the link from trait self‐control to job performance, we ran bootstrapping procedures with 5000 iterations using the *lavaan* package in R (Rosseel, 2012). We included the two strategies identified to predict enhanced performance in the multiple regression (i.e., situational modification of somatic conditions and autonomous motivation) as parallel mediators of the relationship between t_1_ trait self‐control and t_2_ performance, again controlling for t_1_ performance. Results are summarised in Figure 2. The total indirect effect was significant (*β* =  0.17, 95% confidence interval [CI] [0.06, 0.32]) and accounted for 77.46 per cent of the total effect. Situation modification regarding somatic conditions (61.21%) accounted for a relatively larger share of the total indirect effect than autonomous motivation (38.79%).^6^ In sum, Hypothesis 4 was partially supported.

### Supplemental analysis

Our preregistration included subjective well‐being as another important outcome variable besides job performance. Due to space constraint, we only summarise key findings; full results can be found online (https://osf.io/9egkb/). As expected, trait self‐control negatively predicted burnout (*b* = −0.50, *p* = .002). From all self‐control strategies, only autonomous motivation showed a significant association with burnout (*β* = −0.56, *p* < .001) and mediated the self‐control‐burnout link (indirect effect: *β* = −0.12, 95% CI [−0.22, −0.04]). These findings (although not at the heart of the present work) suggest that autonomous motivation is important for employees' well‐being and thereby extend past research on job autonomy (Shirom et al., 2010) to corresponding motivational aspects.

## GENERAL DISCUSSION

A plethora of employees around the globe have experienced manifold challenges to maintain their job performance while working from home during the COVID‐19 crisis. Many of these challenges pertain to self‐control. To date, it was largely unknown how employees deal with such pronounced self‐control demands. Addressing this question, we explored which self‐control strategies employees report to use and examined the role of these strategies in predicting telework outcomes. Further, we examined whether these self‐control strategies can explain the link between employees' trait self‐control and their job performance while working from home.

Results of our qualitative analysis show that employees report a variety of strategies to work productively from home. The strategies can be classified as targeting situation modification to change the circumstances of the given situation (i.e., altering physical, somatic, and social conditions) and cognitive change to think about a given situation differently (i.e., goal setting, planning/scheduling, and autonomous motivation). Of these strategies, altering the physical environment and planning/scheduling were named most often, whereas altering social conditions and enhancing autonomous motivation were reported infrequently.

With our preregistered quantitative analyses, we built upon and advanced these qualitative findings in three ways. First, we examined whether employees' trait self‐control measured at the beginning of the COVID‐19 lockdown predicts the use of self‐control strategies 1 month later. Second, we tested whether (and how) these self‐control strategies are related to teleworking performance. Third, we tested whether (and which) self‐control strategies can empirically explain the self‐control‐performance link (see de Ridder et al., 2012). Our mixed‐methods approach has meaningful implications for the self‐control literature and its application in organisational psychology. We show that employees higher in trait self‐control are indeed more likely to use the strategies established in our qualitative analysis (the only exception being modification of social conditions). Our results also show that not all of these strategies—let alone the most prevalent ones—are associated with higher performance. Indeed, only modifying somatic conditions and autonomous motivation were linked to higher performance. In sum, we found that employees high in trait self‐control changed their somatic conditions to their advantage (e.g., put on some fresh clothes), which was also linked to higher performance while working from home. Further, they were more autonomously motivated (e.g., enjoyed working from home), which was also linked to better performance (and less burnout).

### Contributions to theory and research

Past research has emphasized the beneficial effects of trait self‐control on work‐related outcomes (e.g., Cohen et al., 2014; Converse et al., 2012). The present study corroborates these findings in showing that employees higher in trait self‐control also show higher job performance while working from home in times of crisis—despite a variety of situational demands. Thus, the present research identifies a crucial psychological antecedent, an individual characteristic that is essential to work productively from home (Kramer & Kramer, 2020), even (or particularly) in times of unexpectedly pronounced demands.

Our mixed‐method approach to elaborate employees' use of self‐control strategies expands the theoretical understanding of the processes by which employees manage to stick to their work‐related goals more effectively and the different mechanisms that underlie positive effects of trait self‐control. Thus far, organisational research has focused on self‐control failures due to limited self‐control capacity; yet “overly focusing on resource depletion while trying to study self‐control is akin to overly focusing on a single tree while trying to study a forest” (Lian et al., 2017, p. 717). Our findings establish other relevant trees in this self‐control forest that are worth paying attention to. Specifically, employees higher in trait self‐control employed more proactive strategies (i.e., changing somatic conditions) or cognitively shaped their thoughts to a given situation (i.e., foster autonomous motivation). These strategies were also linked to higher job performance. Both strategies are deployed comparably early in the process of enacting self‐control (Duckworth et al., 2014); thus, they may preempt situations in which employees have to effortfully inhibit undesirable behavior. In terms of our introductory example, the impulse to relax on the nearby sofa may be less pronounced (or even absent) when the employee has slept sufficiently and emphasizes autonomous reasons for mastering a current task.

Our findings contribute to the process model of self‐control (Duckworth et al., 2014) in three ways. First, our qualitative analysis of 480 open responses indicated that the general strategy of situation modification can be further divided into three substrategies—modifying physical, somatic, and social conditions. This nuanced perspective expands prior empirical research that focused predominantly on individuals removing temptations to prevent distractions (i.e., physical conditions; e.g., Duckworth, White, et al., 2016; Ent et al., 2015). Our quantitative results show that although individuals indeed use all three situation modification strategies and trait self‐control predicts the use of all three, only changing somatic conditions—but not physical or social conditions—was positively associated with teleworkers' job performance. Thus, the present findings challenge the existing focus of previous research on modifying physical conditions. Future research should consider all three forms as distinct and important aspects of situation modification. Based on our findings, this future research should delve deeper into the processes by which modifying somatic conditions (e.g., appropriate clothes, sleep, and fresh air) fosters job performance. For instance, having slept sufficiently might lead employees to have more energy (Weigelt & Prem, 2021) and to perceive their work as less demanding (e.g., Casper & Wehrt, 2021), thus being better able to perform well. But why does modifying somatic conditions foster job performance more effectively than changing physical or social conditions? We suggest that somatic conditions are the aspect of a situation that teleworkers are most capable of changing to their advantage. In contrast, advantageously modifying social conditions is hampered by lockdown measures that aim at limiting social contacts, and physical conditions often depend on employees' telework arrangement and given possibilities at home. Thus, teleworkers may particularly draw from changing the somatic conditions to their advantage—a strategy that is relatively easy to implement in telework.

Second, our qualitative results showed that employees report to actively foster their autonomous motivation. In this regard, our quantitative results corroborate previous findings that individuals high in trait self‐control show higher levels of autonomous motivation (Converse et al., 2019), as well as extend these findings by showing that autonomous motivation further explains why individuals high in trait self‐control show higher performance. Future research should examine the underlying processes more deeply. There is empirical precedent that being autonomously motivated leads individuals to perceive their work tasks as easier to achieve (Werner et al., 2016) while encountering fewer obstacles (Milyavskaya et al., 2015). Further, the literature on proactivity (e.g., Ohly & Venz, 2021; Parker et al., 2010) and especially job crafting (e.g., Zhang & Parker, 2019) suggests that autonomously motivated employees might be more likely to proactively change their work demands. Thus, future research should investigate whether autonomous reasons to pursue work tasks affects employees' perceptions and/or proactive crafting of demands at their workplace, which, in turn, might enable employees to attain their work‐related goals more effectively.

Third, the process model of self‐control (Duckworth, Gendler, & Gross, 2016) has suggested that situation modification strategies are more effective than other self‐control strategies as they intervene earliest in the process of developing (potentially undesirable) impulses. Indeed, individuals rate strategies deployed early in the process (e.g., situation modification) as markedly more effective than strategies deployed later (e.g., cognitive change; Duckworth, White, et al., 2016). Direct evidence for this theoretical assumption is quite heterogeneous, however. Whereas some experimental research found situational strategies to be more effective than response modulation or no strategy at all (Duckworth, White, et al., 2016), others showed strategies at different stages as being similarly effective (Milyavskaya et al., 2020; Williamson & Wilkowski, 2020) or report mixed results (Hennecke et al., 2019). By investigating different strategies in their association with teleworkers' performance, we contribute to this recent discussion. In line with the latter finding, we found one specific situation modification strategy (somatic conditions) and one cognitive change strategy (autonomous motivation) to be linked to improved performance while working from home. This result enriches ongoing discussions that the effectiveness of self‐control strategies might depend on a variety of factors (see Hennecke & Bürgler, 2020). For example, a recent study found that using multiple self‐control strategies is particularly effective (Milyavskaya et al., 2020); yet our study suggests that indeed the quality of self‐control strategies (also) plays an important role. Our findings point to valuable avenues for future research that might further examine moderators of the strategies' effectiveness and the joint role and interplay of multiple strategies (see Ford et al., 2019, for a similar reasoning with respect to emotion regulation). Regarding the latter, we deem person‐centered analysis a particularly promising approach for future research to examine which intraindividual combination of strategies is most beneficial (e.g., Gabriel et al., 2020).

### Practical implications

The broad shift to working from home during the COVID‐19 pandemic unveiled numerous advantages of teleworking arrangements. As a result, employees wish for (Stürz et al., 2020) and employers plan on (FAZ, 2020) respective arrangements after the pandemic. At the same time, working from home poses particular demands on employees that might impede their performance. In this regard, our findings offer valuable applied implications for the future of work. Specifically, work‐from‐home arrangements indeed seem to “require selection of workers who are better suited to work from home, training of such workers on more efficient methods of remote work, and greater monitoring of the quality and productivity of those assigned to work from home” (Kramer & Kramer, 2020, p. 2). The present findings establish trait self‐control as a key psychological antecedent, a crucial interpersonal characteristic that distinguishes individuals who are better equipped to work productively from home from others who likely struggle more. In this regard, prior research suggests that repeatedly practicing self‐control can further improve one's trait self‐control (see Friese et al., 2017, for a meta‐analyis). Hence, trainings on self‐control and different self‐control strategies may be beneficial. Regarding the latter, we identified modifying somatic conditions and autonomous motivation as self‐control strategies that were crucial for employees' improved performance while working from home.

### Limitations

We wish to transparently highlight relevant limitations of our study. First, although we realised two waves of data collection that enabled us to consider change in performance, our design does not allow to draw strong causal inferences, particularly for the mediation findings (Spencer et al., 2005). As such, we cannot determine whether situation modification of somatic conditions and autonomous motivation represent true causes of teleworkers' job performance. With this in mind, implementing trainings that aim to improve individuals' use of self‐control strategies is not only exciting from a practical point of view. By conducting such intervention studies (Fiedler et al., 2011), future research could provide further insights on the causality of the link between self‐control, self‐control strategies, and employees' performance.

Second, we wish to urge caution to not overinterpret nonsignificant findings with regard to the self‐control strategies. As outlined before, past research has produced empirical support for the effectiveness of some of these strategies, for instance, situation modification of physical conditions (Duckworth, White, et al., 2016) and goal setting (Epton et al., 2017). In the present study, nonsignificant findings may also result from true effects being too small to be detected with our sample size (*N* = 106). Hence, the most effective self‐control strategies were supported in the present data; yet it may be premature to dismiss the others completely. Although our sample may lack the statistical power to detect potentially (very) small effects, we wish to note that our diverse, heterogeneous sample of experienced employees recruited via established professional networks is likely advantageous and more informative than less diverse samples recruited through crowdsourcing platforms (Peer et al., 2017).

Third, the present analyses relied on self‐report measures, which, in general, may lead to common method bias (Podsakoff et al., 2012). We established several countermeasures to minimize this bias: We measured trait self‐control weeks before job performance, we controlled for t_1_ performance when predicting t_2_ performance, and social desirability did not markedly affect the results. Besides, if effects were driven solely by common method bias, more (if not all) of the self‐control strategies should have shown positive effects and/or mediated the self‐control‐performance link. Notably, prior research has established that performance ratings by employees and supervisors overlap (Heidemeier & Moser, 2009), possibly with more lenient self‐ratings.

Fourth, we need to discuss limitations with regard to the employed measures. We assessed some of the self‐control strategies with one‐item or two‐item measures. The follow‐up validation study empirically corroborated that these items adequately captured the respective self‐control strategy (as indicated by uniform factor loadings and bivariate correlations). Nevertheless, future research should assess self‐control strategies using multi‐item measures as they often are more reliable and outperform single‐item measures in their predictive validity (Diamantopoulos et al., 2012). The items used in the validation study might function as a starting point in this endeavor.

Finally, with regard to shortcut strategies, the time to develop habits in the exceptional situation of working from home during the COVID‐19 crisis may have been too short. Indeed, prior research suggests that whereas some individuals manage to form habits within 18 days, others need half a year (Lally et al., 2010). Hence, after 1 month into lockdown, employees may not have sufficiently established their supporting habits yet. By now, after months of lockdown experience, teleworking employees may rely more strongly on established habits to attain their work‐related goals (e.g., Galla & Duckworth, 2015).

### Conclusion

The COVID‐19 crisis turned the world of work upside down. The sudden shift to working from home has confronted employees with unprecedented and unique challenges of navigating the work–nonwork interface. In the present work, we examined which employees are better able to deal with the work‐from‐home experiment and how they do so. The results extend previous research in establishing (a) trait self‐control as a key psychological trait that is essential to work productively from home and (b) self‐control strategies to explain this link from trait self‐control to job performance. The present study and results seek to offer valuable impulses for future research to illuminate the various means by which employees deal with pronounced self‐control demands.

## CONFLICT OF INTEREST

The authors declare that they have no conflict of interest.

## ETHICS STATEMENT

The present research is in line with the ethical guidelines of the German Psychological Society.

## Data Availability

The data and an analysis script that support the findings of this article are openly available on the Open Science Framework at https://osf.io/9egkb/, https://doi.org/10.17605/OSF.IO/9EGKB.

## ENDNOTES

^1^
In the literature, the terms “self‐control” and “self‐regulation” are often used interchangeably (Johnson et al., 2018). We will use the more established term “self‐control”, but we indeed refer to the broad range of possible means by which individuals' make themselves initiate or persist in (potentially aversive) activities, not only via effortful control over impulses.

^2^
For the quantitative approach, we planned our required sample size a priori and additionally conducted sensitivity analyses based on the final sample of 106 participants. For a mediation effect of small‐to‐moderate size (*β* = 0.26) and a bias‐corrected bootstrap procedure striving for a power of .80, 148 participants are needed (Fritz & MacKinnon, 2007; Götz et al., 2021). Due to dropouts, our final sample size was 106. Sensitivity analysis for a multiple regression with seven predictors with *α* = .05 shows that this sample had an 80% chance to detect an effect of *f* ^2^ = 0.15 and a 95% chance to detect an effect of *f* ^2^ = 0.22.

^3^
For our self‐developed scale on goal setting, we found a markedly higher Coefficient *H* compared with Cronbach's *α*. To account for the fact that each item can contribute different amounts of information to the overall scale (i.e., *λ*_1_ = .85, *λ*_2_ = .80, and *λ*_3_ = .43), we additionally created an optimally weighted scale by extracting factor scores. Using the optimally weighted factor score for goal setting in all analyses led to similar patterns of results as reported in the text. Additionally, we ran this robustness analysis using optimally weighted factor scores for all measures with more than two items. Again, this led to the same pattern of results, with trait self‐control predicting all self‐control strategies (except for modifying social conditions) and modification of somatic conditions and autonomous motivation mediating the self‐control‐performance link.

^4^
Following our preregistered analysis plan (https://osf.io/yw8ma), we only control for situational demands in our first hypothesis, which investigates the association between trait self‐control and job performance, above and beyond these situational demands. However, controlling for situational demands in further analysis also led to a similar pattern of results as reported in the text.

^5^
In the validation study (*N* = 174), we found the same pattern of results. Trait self‐control was associated with each self‐control strategy (0.18 < *β* < 0.51 and .015 < *p* < .001) except for altering social conditions (*β* = 0.13, *p* = .082). These findings again corroborate that the reported single‐item measures adequately captured each self‐control strategy.

^6^
We additionally conducted these mediation analyses while entering all self‐control strategies as parallel mediators into the mediation model. Corroborating the multiple regression results, only (a) situation modifications of somatic conditions and (b) autonomous motivation showed a significant association with performance (b‐paths). To attain higher statistical power, we constrained our analyses and results to the more parsimonious mediation model with only two mediators (Götz et al., 2021).
